# Ultrasonic assessment of liver stiffness and carotid artery elasticity in patients with chronic viral hepatitis

**DOI:** 10.1186/s12876-018-0910-z

**Published:** 2018-12-05

**Authors:** Jing-Hua Li, Ning Zhu, Ying-Bin Min, Xiang-Zhou Shi, Yun-You Duan, Yi-Lin Yang

**Affiliations:** Department of Ultrasound Diagnosis, Tangdu Hospital, Fourth Military Medical University, Xi’an, 710038 Shaanxi Province China

**Keywords:** Ultrasound, HBV, HCV, Carotid artery elasticity

## Abstract

**Background:**

This study investigated the relationship between liver stiffness and carotid artery elasticity in patients with chronic viral hepatitis. We used an acoustic radiation force impulse (ARFI) technique to measure stiffness, and a radio frequency (RF) vascular quantitative ultrasound technique to measure changes in common carotid artery elasticity and vascular function.

**Methods:**

Two-hundred seventeen patients with chronic viral hepatitis caused by either hepatitis B virus (HBV) or hepatitis C virus (HCV) were enrolled. We divided the patients into two groups, one comprising 147 patients with chronic hepatitis B (CHB) (98 men and 49 women, average age 46.5 ± 12.2 years) and another comprising 70 patients with chronic hepatitis C (CHC) (47 men and 23 women, average age 47.6 ± 12.1 years). Additionally, 64 healthy age- and sex-matched participants (43 men and 21 women, average age 47.8 ± 5.1 years) were selected as the control group. The ARFI technique was used to measure liver stiffness and the RF ultrasound technique was used to measure carotid artery elasticity parameters including intima-media thickness (IMT), pulse wave velocity (PWV), arterial wall dilation coefficient (DC), compliance coefficient (CC), sclerosis indices α and β, and augmentation index (Aix). Clinical indicators, liver stiffness, and carotid artery elasticity parameters were observed and compared between the different age groups to investigate the correlation between carotid artery elasticity parameters and liver stiffness.

**Results:**

The ARFI values for the CHB and CHC groups were significantly higher than those for the control group (1.84 ± 0.52 vs. 1.04 ± 0.11 m/s; 1.86 ± 0.37 vs. 1.04 ± 0.11 m/s, respectively; *P* < 0.001). When compared to the control group, both CHB and CHC groups showed an IMT of the same order, but had significantly higher elasticity parameters, such as α and β, as well as lower DC and CC values (*P* < 0.001). The PWV of the CHC group was significantly higher than that of the control group (7.98 ± 1.42 vs. 6.09 ± 0.90 m/s, *P* < 0.001). In the CHB group, all parameters including ARFI, IMT, PWV, DC, CC, α and β, were significantly different between the two age groups (*P* < 0.05). Within the CHC group, all parameters including IMT, PWV, DC, α and β, were significantly different between the two age groups (*P* < 0.05), except for ARFI, wherein the difference was not statistically significant. The correlation analysis and stepwise multiple linear regression analysis indicated that for patients with CHB, age was an independent predictor of common carotid artery IMT (R^2^ = 0.468, F = 54.635, and *P* < 0.001). For patients with CHC, age and blood sugar were independent predictors of common carotid artery IMT (R^2^ = 0.465, F = 29.118, and *P* < 0.001).

**Conclusion:**

Although based on ARFI and RF ultrasound, the carotid artery IMT in patients with CHB and CHC was not significantly higher than that in the control group, their functional elasticity parameters had already changed. This finding serves as a useful reference for the clinical diagnosis of vascular diseases in patients with viral hepatitis.

**Trial registration:**

ClinicalTrials: ChiCTR1800015859 25/04/2018.

## Background

Vascular wall elasticity is an important indicator of abnormal lipid metabolism in vascular disease, which is caused by pathogen-mediated chronic liver damage [1, 2]. Signals in the radio frequency (RF) vascular quantitative ultrasound technique quantify the intima-media thickness (IMT) and arterial stiffness within blood vessels and can serve as a sensitive indicator of early changes in vascular wall stiffness. The progression of liver fibrosis changes liver morphology and hepatic haemodynamics and decreases liver function. Liver biopsy has been the gold standard to measure and classify liver fibrosis, but because of its invasiveness, its clinical use is limited. Recently, elastography, a novel non-invasive technique for evaluating the degree of liver fibrosis, has gradually been applied in clinical practice and has been included in several liver disease diagnosis and treatment guidelines [3]. Preliminary experiments found that carotid artery elasticity parameters in patients with coronary artery disease and diabetes differed from those of healthy subjects [4]. However, whether these parameters were correlated with the degree of liver fibrosis in patients with chronic viral hepatitis have not been not studied using the new vascular measurement technologies. As a result, we conducted relevant clinical investigations and experiments to understand the extent of the changes in carotid artery morphology and function in patients with chronic viral hepatitis. These findings would help to determine whether the liver fibrosis is related to macrovascular diseases.

## Methods

### Research targets and grouping

A total of 217 consecutive patients with chronic viral hepatitis that were treated at our hospital between December 2015 and March 2017 were enrolled. The study population comprised 147 patients with Chronic hepatitis B (CHB) and 70 patients with Chronic hepatitis C (CHC). Patients were admitted into the CHB group according to the standard chronic hepatitis B diagnostic criteria [5], defined as hepatitis B surface antigen-positive without detectable ascites. Decompensated patients with symptoms such as ascites and lower oesophageal varices were excluded from this group. In the CHB group, there were 98 men and 49 women, with an average age of 46.5 ± 12.2 years. Among them, 34 patients had biopsy-proven liver fibrosis. Patients enrolled in the CHC group were hepatitis C virus antibody- (HCVAb) positive and had decompensated liver function. The exclusion criteria for the CHC group were the same as those for the CHB group. The CHC group consisted of 47 men and 23 women, with an average age of 47.6 ± 12.1 years. Among them, a biopsy confirmed liver fibroses in 21 patients. Contemporaneously, 64 healthy subjects comparable in age and sex, which was defined by no history of liver disease, hepatitis B virus surface antigen (HBsAg) and HCVAb negativity, normal haemogram, liver, and kidney laboratory examinations, and no detection of liver diseases via 2-D ultrasound examination. Patients with disorders, such as high blood pressure, hypercholesterolemia, and dyslipidemia were excluded. The control group included 43 men and 21 women, with an average age of 45.8 ± 10.6 years.

### Instruments and methods

#### Liver Elastography ultrasound

**Fig. 1:**
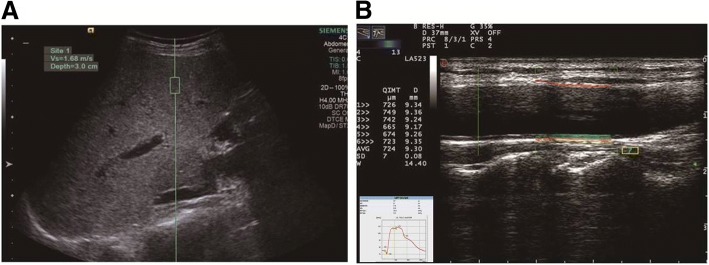
ARFI liver stiffness images and common carotid artery RF ultrasound images in patients with CHB: **a** Use of the ARFI technique to measure liver stiffness in patients with CHB using a conventional ultrasound interface. **b** Use of QIMT and QAS techniques to quantify the carotid artery intima-media thickness (IMT) and vascular elasticity in patients with CHB

The acoustic radiation force impulse (ARFI) technique uses the 4C1 convex probe from the Color Doppler Diagnostic Ultrasound Scanner (Siemens, Siemens Acuson S2000) with a frequency of 3.0 to 4.5 MHz. During ARFI imaging, measurements were performed with the probe placed between the rib bones with the patient lying in either a dorsal decubitus or left recumbent position, taking normal breaths under a resting state or holding their breath, with displays of real-time 2-D images of the right liver lobe. When the image became clear, the operator used the cursor to locate a 5 mm × 10 mm-sized region of interest that targeted the liver parenchyma area and was free of vessels and bile duct. The measurement depth was fixed at 3 cm. When echoing was uniform within the sampling region, the operator pressed the probe button to freeze the image and display the depth and shear wave velocity (in m/s) in the region of interest. This measurement procedure was repeated 10 times for each patient. The median value of the measurements was recorded as the final result [6] (Fig. 1a).

#### Quantitative measurement of the common carotid artery

Measurements of the common carotid artery were obtained using the LA523 vascular probe from the Esaote Mylab Color Doppler Diagnostic Ultrasound Scanner with a frequency of 4 to 13 MHz. The ultrasound scanner was equipped with the RF-data technique and the Mylab Desk analysis working station. To take quantitative measurements of the common carotid artery, the patient was placed in the dorsal decubitus position, and instructed to breath normally in a resting state. After the patient’s systolic and diastolic blood pressures in the right upper limb were measured, the patient’s neck was sufficiently exposed. The ultrasonic probe was moved down longitudinally from the beginning of the common carotid artery, skipping the bifurcation area by 1 cm and the plaque sites. The operator then moved the sampling frame to the region for measurement, and the scanner automatically recorded the IMT and the elasticity parameters during 6 cardiac cycles. When the standard deviation of the IMT was less than 30, the indicator would turn green, suggesting that the measurements were stable. At that time, the values of parameters such as IMT, pulse wave velocity (PWV), arterial wall dilation coefficient (DC), arterial wall compliance coefficient (CC), sclerosis indices α and β, and augmentation index (Aix) were exported as the final results [7] (Fig. 1b).

#### Clinical information

For all subjects, the following information was collected: (1) laboratory indicators including blood sugar, glycated haemoglobin, total cholesterol, triglycerides, low-density lipoprotein, high-density lipoprotein, alanine aminotransferase, aspartate aminotransferase, albumin, globulin, and platelets. (2) blood pressures including systolic and diastolic blood pressures.

### Statistical analysis

Statistical analysis was performed using SPSS statistical software (version 19.0, IBM Corp., Armonk, NY, USA). Metrological data were expressed as average ± standard deviation ($\overline{X}$ ± S), while classification data were expressed as percentages (%). Inter-group measurement data were compared using one-way analysis of variance (ANOVA) and Levene’s homogeneity of variance test. The average values of the two groups of measurement data were compared using the independent sample t test. A determination of correlation was conducted using Pearson’s linear correlation method. Casual relationships of various intra-group parameters were investigated using either linear regression analysis or stepwise multiple linear regression analysis.

## Results

### General clinical data of the subjects

General clinical data for all study participants are listed in Table 1. We observed that there was no significant difference in age or sex among the three groups. Compared to the control group, the CHB group demonstrated a higher blood sugar level (5.3 ± 1.1 vs. 4.9 ± 0.5 mmol/L; *P* = 0.010) as well as higher levels of other parameters such as platelets, albumin, AST and ALT, whereas the systolic and diastolic blood pressures were similar. The CHC group demonstrated a higher blood sugar level (5.3 ± 1.1 vs. 4.9 ± 0.5 mmol/L, *P* < 0.001), higher glycated haemoglobin level (6.8 ± 1.6 vs. 5.3 ± 0.5%, *P* < 0.001), and higher levels of other parameters such as AST, ALT, and platelets compared to the control group. However, there was no significant difference in the cholesterol, triglycerides, HDL, and LDL levels.

**Table 1:** General clinical data of all study participants

	Control Group *n* = 64	Patients with chronic viral hepatitis		F	*P*
		Patients with CHB *n* = 147	Patients with CHC *n* = 70		
Age, Years	45.8 ± 10.6	46.5 ± 12.2	47.6 ± 12.1	0.400	0.670
Men Percentage, Count (%)	43(67)	98(67)	47(67)	–	–
Systolic pressure, mmHg	115.7 ± 11.6	116.2 ± 13.1	119.7 ± 12.8	2.166	0.177
Diastolic, pressure mmHg	71.2 ± 8.7	73.2 ± 8.6	71.2 ± 8.6	1.872	0.156
Blood Glu, mmol/L	4.9 ± 0.5	5.3 ± 1.1	6.0 ± 1.7	15.933	< 0.001
Glycated haemoglobin, %	5.3 ± 0.5	5.5 ± 1.4	6.8 ± 1.6	30.804	< 0.001
BMI, Kg/m^2^	23.2 ± 2.6	23.5 ± 3.0	24.4 ± 3.4	2.936	0.055
Cholesterol, mmol/L	3.8 ± 1.1	3.8 ± 1.3	3.7 ± 1.1	0.373	0.689
Triglycerides, mmol/L	1.2 ± 0.7	1.2 ± 0.5	1.2 ± 0.6	0.185	0.831
LDL, mmol/L	1.9 ± 0.8	1.8 ± 0.8	1.7 ± 0.8	0.695	0.500
HDL, mmol/L	1.0 ± 0.3	1.2 ± 0.4	1.1 ± 0.5	3.115	0.046*
Platelets, 10^9^ /L	187.6 ± 70.2	137.4 ± 77.8	138.3 ± 64.3	11.598	< 0.001
AST, U/L	23.8 ± 8.7	45.8 ± 34.2	37.7 ± 24.6	13.927	< 0.001
ALT, U/L	28.5 ± 13.8	46.1 ± 38.5	43.1 ± 40.4	5.765	0.004
Albumin, g/L	43.8 ± 7.4	40.9 ± 8.9	40.8 ± 8.2	3.104	0.046*
Globulin, g/L	24.2 ± 6.3	25.3 ± 7.2	24.9 ± 7.1	0.566	0.569

Note: *CHB* chronic hepatitis B, *CHC* chronic hepatitis C, *BMI* Body mass index, *LDL* Low-density lipoprotein, *HDL* High-density lipoprotein, *AST* Aspartate aminotransferase, *ALT* Alanine aminotransferase. * *P* < 0.05 when compared to the control group

### Comparison of liver stiffness and elasticity parameters among groups

**Fig. 2:**
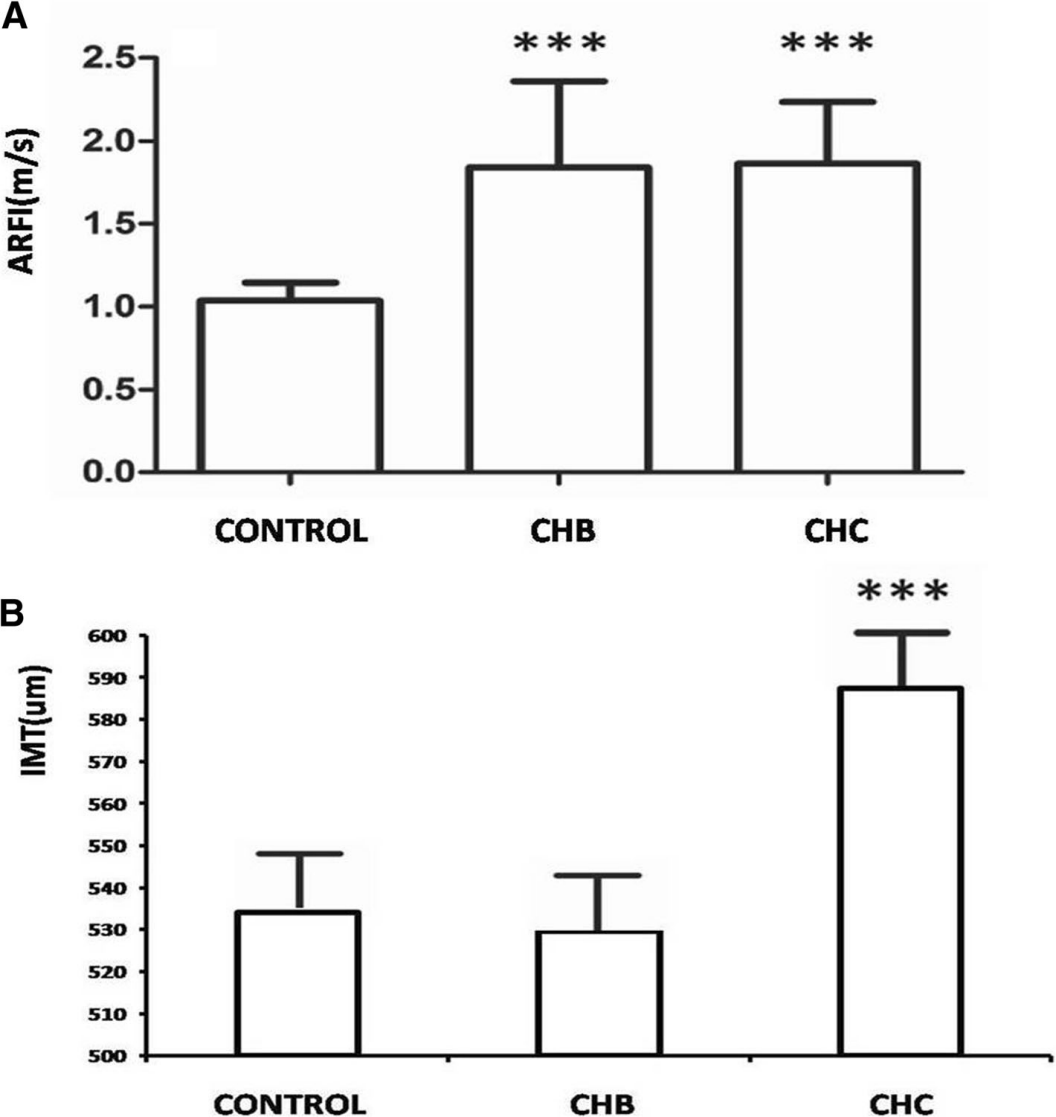
Comparison of liver stiffness and carotid artery IMT values among the 3 groups: **a** Use of the ARFI technique to measure liver stiffness in patients with CHB, CHC and control group using a conventional ultrasound interface. **b** Use of QIMT and QAS techniques to quantify the carotid artery intima-media thickness (IMT) and vascular elasticity in patients among the 3 groups. Note: ARFI: acoustic radiation force impulse; IMT: intima-media thickness; CHB: chronic hepatitis B; CHC: chronic hepatitis C; ***: when compared to the control group, *P* < 0.001

Results measured by the ARFI technique showed that both the CHB group (1.84 ± 0.52 m/s) and the CHC group (1.86 ± 0.37 m/s) had significantly higher liver elasticity parameters than the control group, and the inter-group differences were statistically significant (F = 90.806, *P* < 0.001) (Fig. 2a).

Comparison of carotid artery IMT values measured by RF ultrasound showed statistically significant differences (*P* = 0.015) between the control group (534.08 ± 134.25 μm), the CHB group (529.56 ± 131.04 μm), and the CHC group (587.34 ± 162.70 μm). An intergroup comparison showed significant difference between the CHB and the CHC group (*P* = 0.032) (Fig. 2b).

**Fig. 3:**
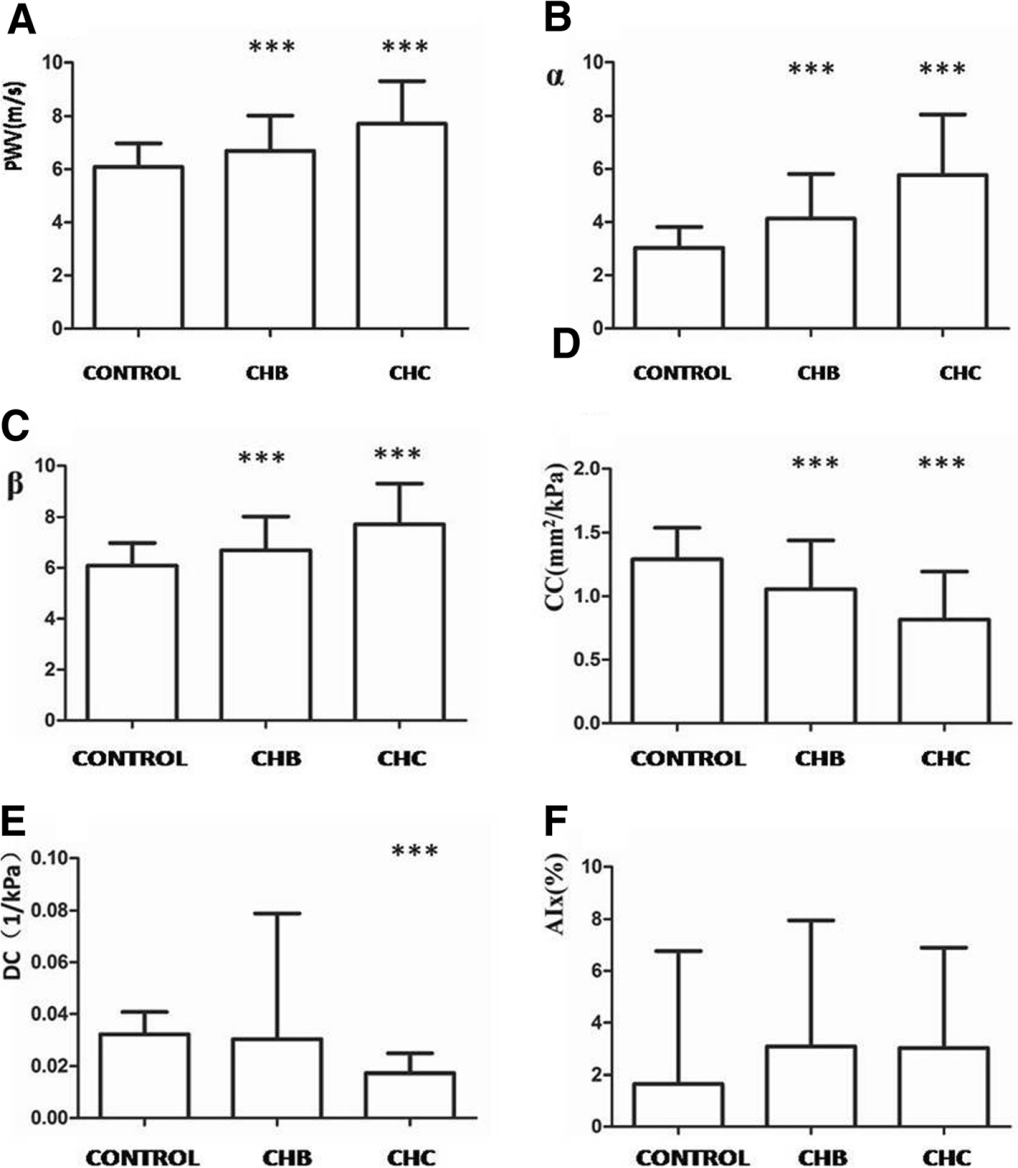
Carotid elasticity parameters and comparisons among the 3 groups: **a** PWV values and comparisons among the 3 groups; **b** α values and comparisons among the 3 groups; **c** β values and comparisons among the 3 groups; **d** CC values and comparisons among the 3 groups; **e** DC values and comparisons among the 3 groups; **f** Aix values of the 3 groups. CHB: chronic hepatitis B; CHC: chronic hepatitis C; PWV: pulse wave velocity; CC: arterial wall compliance coefficient; DC: arterial wall dilation coefficient; Aix: augmentation index. Note: ***: when compared to the control group, *P* < 0.001

The PWV measurements of the CHC group (7.98 ± 1.42) were evidently higher than those of the CHB and the control groups, which were 6.70 ± 1.32 and 6.09 ± 0.90 m/s respectively. Differences in the intergroup comparisons were statistically significant (F = 40.310, *P* < 0.001) (Fig. 3a). The α values of the CHC and the CHB group were significantly higher than those of the control group, which were 3.03 ± 0.79, 4.13 ± 1.68, and 5.77 ± 2.29, respectively, with statistically significant differences under intergroup comparisons (F = 44.036, *P* < 0.001) (Fig. 3b). Similarly, the β values of the CHC and the CHB group were significantly higher than those of the control group, which were 6.17 ± 1.58, 8.42 ± 3.37, and 11.67 ± 4.53, respectively, with statistically significant differences under intergroup comparisons (F = 44.493, *P* < 0.001) (Fig. 3c). On the contrary, the DC and CC values of the CHC and CHB group were significantly lower than those of the control group. The DC values of the three groups were 0.32 ± 0.008, 0.030 ± 0.008, and 0.017 ± 0.008 1/kPa, respectively (Fig. 3e), with statistically significant intergroup differences (F = 3.897, *P* = 0.021). The CC values were 1.290 ± 0.248, 1.054 ± 0.385, and 0.815 ± 0.378 mm^2^/kPa, respectively (Fig. 3d), with statistically significant intergroup differences (F = 29.717, P < 0.001). The DC and CC values of the CHC group were also significantly lower than those of the CHB group (*P* < 0.001). Aix measurements showed no statistically significant differences among the 3 groups (F = 2.237, *P* = 0.0109) (Fig. 3f).

### Comparison of liver stiffness and left common carotid elasticity parameters among different age groups

All subjects were divided into two groups according to their age (Tables 2 and 3). with age 50 years being the dividing line. A comparison of the two groups showed no significant difference in ARFI values. However, in terms of carotid elasticity parameters, except for Aix, all other parameters including IMT, PWV, DC, CC, α, and β were significantly different between the two groups (all *P* values < 0.001). In the CHB group, ARFI values, as well as parameters including IMT, PWV, DC, CC, α, β, and Aix were all significantly different between the two age groups (all *P* values < 0.05). In the CHC group, IMT, PWV, DC, α, β, and Aix were significantly different between the two age groups (all *P* values < 0.05).

**Table 2:** Liver stiffness and left common carotid elasticity parameters in the different age groups

	Age, Year	No.	ARFI, m/s	IMT, μm	PWV, m/s	DC, 1/kPa
Control Group	< 50	41	1.02 ± 0.09	478.44 ± 107.86	5.72 ± 0.73	0.035 ± 0.007
	≥50	23	1.06 ± 0.13	633.26 ± 120.02	6.75 ± 0.80	0.026 ± 0.007
	*P*	–	0.127	< 0.001*	< 0.001*	< 0.001*
CHB Group	< 50	86	1.72 ± 0.50	456.70 ± 87.56	6.26 ± 1.35	0.038 ± 0.062
	≥50	61	2.02 ± 0.50	630.30 ± 112.17	7.32 ± 1.00	0.020 ± 0.007
	*P*	–	< 0.001*	< 0.001*	< 0.001*	0.009*
CHC Group	< 50	41	1.84 ± 0.38	526.63 ± 130.03	7.70 ± 1.57	0.018 ± 0.009
	≥50	29	1.89 ± 0.36	673.17 ± 167.48	8.36 ± 1.09	0.015 ± 0.006
	*P*	–	0.569	< 0.001*	0.041*	0.079

*ARFI* radio frequency, *IMT* intima-media thickness, *PWV* pulse wave velocity, *DC* arterial wall dilation coefficient

* *P* < 0.05 when compared to the control group

**Table 3:** Liver stiffness and left common carotid elasticity parameters in the different age groups

	Age, Year	No.	CC, mm^2^/kPa	α	β	Aix,%
Control Group	< 50	41	1.376 ± 0.259	2.70 ± 0.54	5.54 ± 1.10	0.82 ± 5.18
	≥50	23	1.137 ± 0.129	3.63 ± 0.81	7.30 ± 1.70	3.14 ± 4.76
	*P*	–	< 0.001*	< 0.001*	< 0.001*	0.082
CHB Group	< 50	86	1.139 ± 0.420	3.71 ± 1.69	7.58 ± 3.41	1.55 ± 4.41
	≥50	61	0.935 ± 0.291	4.72 ± 1.51	9.61 ± 2.96	5.25 ± 4.68
	*P*	–	0.001*	< 0.001*	< 0.001*	< 0.001*
CHC Group	< 50	41	0.854 ± 0.423	5.31 ± 1.97	10.65 ± 3.79	1.75 ± 3.45
	≥50	29	0.761 ± 0.303	6.42 ± 2.57	13.11 ± 5.13	4.84 ± 3.75
	*P*	–	0.317	0.044*	0.024*	0.001*

*CC* arterial wall compliance coefficient, *Aix* augmentation index

* *P* < 0.05 when compared to the control group

### Relationship between liver stiffness with carotid artery elasticity in patients with chronic viral hepatitis

**Fig. 4:**
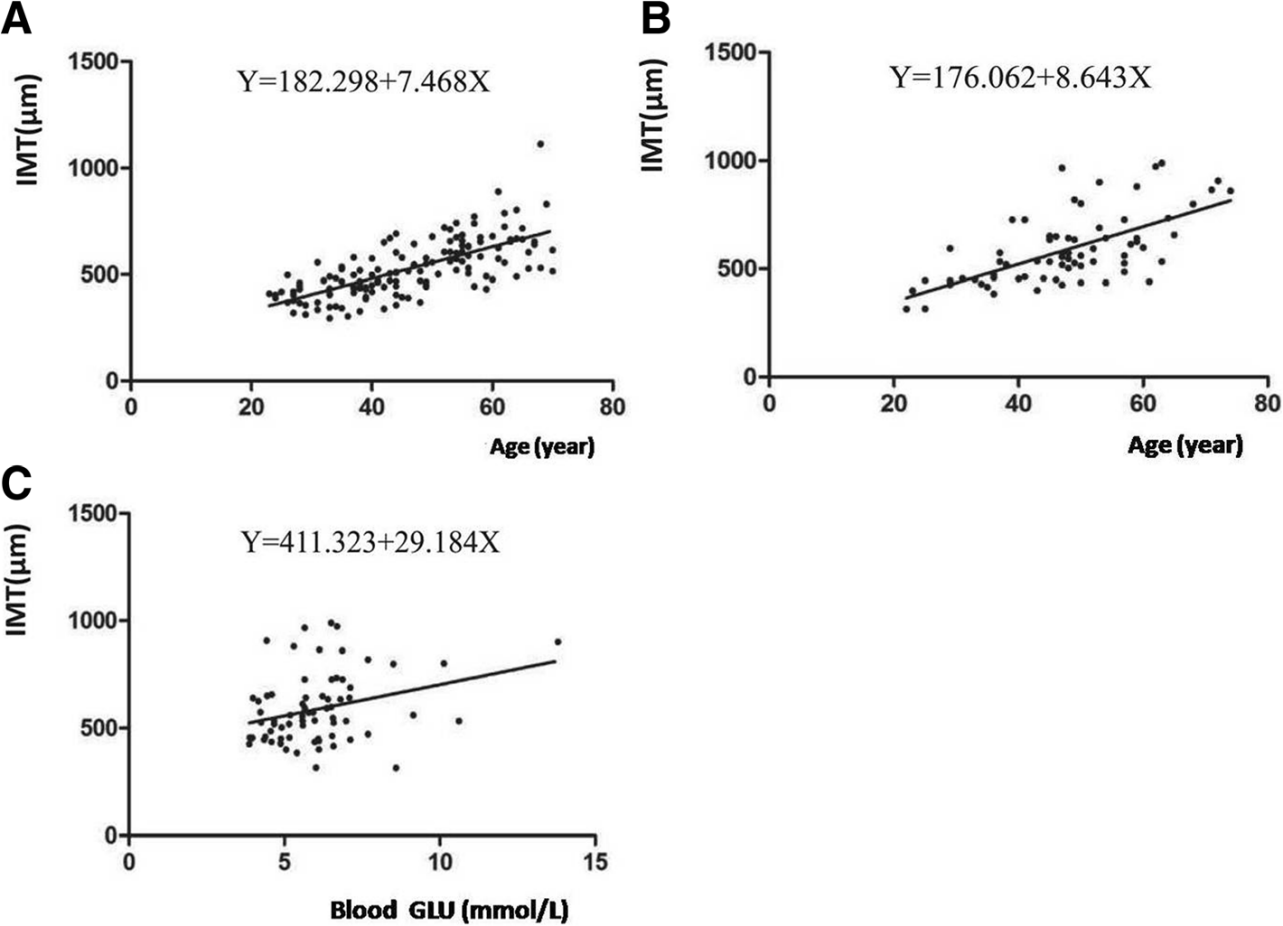
**a** There was a relationship between IMT and age of CHB patients. **b** Relationship between IMT and age of CHC patients. **c** Relationship between IMT and blood glucose meter of CHC patients. Note: IMT: intima-media thickness; CHB: chronic hepatitis B; CHC: chronic hepatitis C

Stepwise multiple linear regression analysis indicated that for patients with CHB, age was an independent predictor of common carotid artery IMT (R^2^ = 0.468, F = 54.635, and *P* < 0.001) (Fig. 4a). For patients with CHC, both age (β = 8.291,*t* = 6.847,P < 0.001) and blood glucose (β = 22.436, *t* = 2/573, *P* = 0.012) were independent predictors of common carotid artery IMT (R^2^ = 0.465, F = 29.118, and *P* < 0.001) (Fig. 4b, c).

## Discussions

Chronic liver disease can cause abnormal lipid metabolism, which, in severe cases, can directly change the peripheral blood vessel walls. Timely and convenient measurements of changes in the peripheral blood vessel walls have a positive effect on preventing detrimental cardiovascular and cerebrovascular events [1, 8]. Previous studies reported that HCV infection can alter in vivo glucose homeostasis and lipid metabolism leading to liver and peripheral insulin resistance [9, 10]. Our study found that patients with CHC not only had thickened IMT compared to the control and the CHB groups, but also had higher PWV, α, and β parameters and lower DC and CC values than the control group. This indicated that both the carotid artery structure and function parameters of the CHC group had changed compared to the control group. This finding was in line with the argument that HCV infection is a risk factor for atherosclerosis [10, 11] . For patients with CHB, although their carotid artery IMT were not thickened, their other parameters such as α and β were higher, and their CC value was lower than those in the control group. These results indicated that, despite normal carotid artery wall structures, their carotid arteries and the elasticity parameters of their carotid artery walls had already changed. A possible explanation is that carotid atherosclerosis could cause both structural and functional changes. One major indicator for structural changes was an increase in the carotid IMT, while functional changes were mainly indicated by changes in carotid artery elasticity [12, 13]. Although structural changes in the carotid artery can cause changes in its elasticity, such elasticity changes may also indicate that IMT thickening is not the only cause of arterial wall composition changes. It is speculated that carotid artery functional changes in patients with CHB may occur before the structural changes. Therefore, this study showed that patients with viral hepatitis maybe suffer a higher risk of cardiovascular events than healthy people, and this finding can provide some reference value for clinical diagnosis and treatment of these patients.

Age is also a critical factor affecting the potential for development of arteriosclerosis [14, 15]. In this study, participants were divided into two groups according to their age, with those aged 50 or above in one group and those aged under 50 in the other. It was found that within the CHB group, the ARFI value (*P* = 0.001) and the carotid artery elasticity parameters (all *P* values < 0.05) differed significantly between the two age groups, indicating that these parameters might be related to the time span of HBV infection. Older patients with chronic hepatitis B are likely to carry the virus for a longer period of time and, consequently, experience a higher degree of liver stiffness. This difference was not observed in the control group, which indicated that aging is not related to the natural aging and fibrosis of the liver. In a study of 459 chronic HBV carriers, liver biopsies showed that the liver tissue inflammatory activity level and degree of liver fibrosis gradually increased with age [16], which was in line with our findings.

However, within the CHC group, the ARFI did not differ significantly between the two age groups (*P* > 0.05). A possible explanation was that patients with CHC have a higher risk of increased blood glucose levels. Studies on using the ARFI technique to grade liver stiffness and fibrosis showed that fat in the liver is an important factor that affects the accurate measurement of the ARFI value [17, 18]. Since 40% of the patients with CHC in this study had elevated blood glucose, the abnormal lipid metabolism caused by abnormal blood sugar levels led to fat deposition in their liver, thus affecting the ARFI values. Although the accuracy of ARFI measurements were affected by abnormal blood glucose contents, measurements of carotid artery elasticity parameters were also found to be significantly abnormal. This indicated that HCV not only significantly affected liver stiffness but also changed carotid artery elasticity. Stepwise multiple linear regression analysis demonstrated that both age and blood sugar are independent predictors of IMT in patients with CHC. Therefore, we speculated that, in addition to lipid metabolism, patients with chronic viral hepatitis also have metabolic syndromes caused by viral infections. The macrovascular damages caused by blood viscosity and hyperglycaemia also affect the structural changes of the carotid artery.

This study was limited by the fact that patients’ diagnoses were made based on clinical diagnosis primarily. Biopsy was used to obtain pathological results in only a limited number of cases. The next step is to obtain results to classify liver fibrosis into different pathological levels, to further exclude any confounding factors.

## Conclusions

In summary, using RF ultrasound and ARFI techniques to measure liver stiffness and carotid artery elasticity in patients with chronic viral hepatitis is beneficial for assessing the liver fibrosis and the structural and functional changes of the carotid artery. This serves as a reference for clinicians to monitor any vascular diseases in these patients.

## Abbreviations

Aix: Augmentation index
ANOVA: Analysis of variance
ARFI: Acoustic radiation force impulse
CC: Compliance coefficient
CHB: Chronic hepatitis B
CHC: Chronic hepatitis C
DC: Arterial wall dilation coefficient
HBV: Hepatitis B virus
HCV: Hepatitis C virus
HCVAb: Hepatitis C virus antibody
IMT: Intima-media thickness
PWV: Pulse wave velocity
RF: Radio frequency
